# Expression of germline markers in three species of amphioxus supports a preformation mechanism of germ cell development in cephalochordates

**DOI:** 10.1186/2041-9139-4-17

**Published:** 2013-06-18

**Authors:** Qiu-Jin Zhang, Yi-Jyun Luo, Hui-Ru Wu, Yen-Ta Chen, Jr-Kai Yu

**Affiliations:** 1 Institute of Cellular and Organismic Biology, Academia Sinica, 128 Academia Road, Section 2, Nankang, Taipei, 11529, Taiwan; 2 Fujian Key Laboratory of Developmental and Neuro Biology, College of Life Sciences, Fujian Normal University, Fuzhou, 350108, People’s Republic of China; 3 Institute of Oceanography, National Taiwan University, No. 1, Section 4, Roosevelt Road, Taipei, 10617, Taiwan

**Keywords:** Cephalochordate, *Nanos*, *Piwi*, Primordial germ cell, *Tudor*, *Vasa*

## Abstract

**Background:**

In a previous study, we showed that the cephalochordate amphioxus *Branchiostoma floridae* has localized maternal transcripts of conserved germ cell markers *Vasa* and *Nanos* in its early embryos. These results provided strong evidence to support a preformation mechanism for primordial germ cell (PGC) development in *B. floridae*.

**Results:**

In this study, we further characterize the expression of *B. floridae* homologs of *Piwi* and *Tudor*, which play important roles in germline development in diverse metazoan animals. We show that maternal mRNA of one of the identified *Piwi-*like homologs, *Bf-Piwil1*, also colocalizes with *Vasa* in the vegetal germ plasm and has zygotic expression in both the putative PGCs and the tail bud, suggesting it may function in both germline and somatic stem cells. More interestingly, one Tudor family gene, *Bf-Tdrd7*, is only expressed maternally and colocalizes with *Vasa* in germ plasm, suggesting that it may function exclusively in germ cell specification. To evaluate the conservation of the preformation mechanism among amphioxus species, we further analyze *Vasa*, *Nanos*, *Piwil1*, and *Tdrd7* expression in two Asian amphioxus species, *B. belcheri* and *B. japonicum*. Their maternal transcripts all localize in similar patterns to those seen in *B. floridae*. In addition, we labeled putative PGCs with Vasa antibody to trace their dynamic distribution in developing larvae.

**Conclusions:**

We identify additional germ plasm components in amphioxus and demonstrate the molecular distinction between the putative germline stem cells and somatic stem cells. Moreover, our results suggest that preformation may be a conserved mechanism for PGC specification among *Branchiostoma* species. Our Vasa antibody staining results suggest that after the late neurula stage, amphioxus PGCs probably proliferate with the tail bud cells during posterior elongation and are deposited near the forming myomere boundaries. Subsequently, these PGCs would concentrate at the ventral tip of the myoseptal walls to form the gonad anlagen.

## Background

For multicellular animals, the segregation of germline cells from somatic cells during embryonic development is an effective way to ensure genome integrity in germ cells. Once segregated, the primordial germ cells (PGCs) are usually kept quiescent to protect the germline genome from potential mutations caused by the differentiation and repeated division of the somatic cells during life [1]. In some developmental model organisms, such as *Caenorhabditis elegans*, *Drosophila*, zebrafish, and *Xenopus*, PGCs are specified early during embryogenesis by the inheritance of maternal determinants [2]. These maternal determinants are usually localized in a specific cytoplasmic region of the egg called germ plasm or nuage, and they consist of mRNAs and proteins from a conserved repertoire of genes, most commonly including *Vasa*, *Nanos*, *Piwi/Ago*, *Tudor*, *Boule/DAZ*, and *bruno* (reviewed in [3,4]). This mechanism of germ cell formation is called preformation mode [5]. In contrast, other animals do not have recognizable germ plasm in the egg, and their germ cells are specified later during development by inductive signals [5]. This type of germ cell development is called epigenesis or induction mode. For example, mouse PGCs are induced in the epiblast by the bone morphogenetic proteins secreted from the extraembryonic ectoderm and visceral endoderm after gastrulation [2]. Notably, in basally branching metazoan animals, such as sponges, hydrozoans, and planarians, germ cells can be generated continuously from adult multipotent stem cell populations [6-8]. Indeed, the same genes (such as *Vasa*, *Nanos*, and *Piwi*) are commonly expressed in both PGCs and multipotent somatic stem cells in various organisms, leading to the hypothesis that PGCs and multipotent somatic stem cells probably share a common ancestry from a population of multipotent progenitor cells and utilize a conserved regulatory gene network for their formation and maintenance [3]. Moreover, the broader phylogenetic distribution of animals that use epigenesis compared with those that use preformation appears to support the idea that epigenesis is probably ancestral [1,5]. However, it should be noted that the designation of the PGC specification mode in some organisms was based on morphological criteria and may not be completely reliable. Indeed, some recent papers using molecular markers or cell lineage-tracing experiments have uncovered the preformation property of germ cell formation in some species that were thought to use epigenesis [9-11]. Thus, many questions remain unresolved regarding the evolution of PGC specification mechanisms, and a reassessment of PGC formation with modern molecular markers in many less well-studied organisms is needed to address these questions.

Opinions on the mechanism of PGC formation in basal chordate amphioxus have been ambiguous in previous literature (reviewed in [5]). Traditional observations using histological approaches or electron microscopy found that the earliest recognizable germ cells are located at the ventral tips of the myomeres in late larvae ([12], reviewed in [13]). Based on these observations, it had been suggested that amphioxus PGCs are derived epigenetically from the existing epithelium of the myocelic walls. However, because these traditional studies mostly focused on adults or larvae, the alternative scenario that the amphioxus PGCs are formed elsewhere in the embryo but later move to the ventral tips of the myomeres, cannot be completely excluded. Notably, using electron microscopy, Holland and Holland [14] identified a region of aggregated vegetal pole cytoplasm, which contains sheets of endoplasmic reticulum associated with electron-dense particles and mitochondria, in fertilized amphioxus eggs and early cleavage-stage embryos. They suggested that this condensed cytoplasm might be the germ plasm in amphioxus. However, the exact developmental fate of this vegetal pole plasm was not investigated for a long time.

In a recent study, we showed that the gene products of germline markers *Vasa* and *Nanos* are localized to the vegetal pole plasm of eggs in Florida amphioxus *Branchiostoma floridae*[11]. After fertilization, these colocalized *Vasa* and *Nanos* maternal transcripts aggregate into a compact granule near the vegetal pole of the embryo and are inherited asymmetrically by a single blastomere during cleavage. Subsequently, this blastomere gives rise to a cluster of cells that display typical characteristics of PGCs. Thus, our results provided strong evidence of a preformation mechanism of PGC development in amphioxus. Furthermore, our findings raised some interesting questions. Is this compact RNA granule in amphioxus equivalent to the germ granules [4] found in other organisms? Are other well-known germ cell-associated components present in this compact RNA granule? Is the preformation mechanism generally used for PGC formation among cephalochordates? How do putative PGCs move from the posterior tail bud region to the gonad anlagen located in the most ventral part of the myomeres in amphioxus larvae?

To address these questions, we analyzed genes encoding Piwi/Ago family proteins and Tudor domain-containing (Tdrd) proteins in amphioxus. In many of the metazoan animals investigated so far (such as *Drosophila*, zebrafish, and mouse), Piwi and Tudor proteins play key roles in germ cell development [15,16]. Piwi proteins belong to a subfamily of the Argonaute (Ago) proteins, which are characterized by two major protein domains: the PAZ and PIWI domains [17,18]. In germ cells, Piwi proteins associate with Piwi-interacting RNAs (piRNAs), which are 24 to 31 nucleotides in length, to target protein-coding transcripts that are associated with germ cell differentiation. Furthermore, Piwi proteins and piRNAs repress the action of endogenous transposable elements to protect the germline genome integrity [19]. The function of Piwi proteins requires the interaction of Piwi proteins with other proteins, and recent studies have identified a group of Tudor domain-containing proteins that interact with Piwi proteins to regulate the Piwi-piRNA pathway for transposon silencing and gametogenesis [16,20]. The Tudor domain is a conserved protein motif found in a wide variety of organisms, including fungi, plants, and animals [21]. Many Tdrd proteins are specifically expressed in the germ cells of metazoan animals and are required for the assembly of the germ granules [15]. In addition, Tdrd proteins serve as docking sites for Piwi proteins to execute their functions and facilitate piRNA biogenesis [15,16,20]. Thus, along with Vasa and Nanos proteins, Piwi and Tdrd proteins play essential roles during germ cell development.

In this study, we identified genes encoding Piwi-like proteins and Tudor domain-containing proteins in the amphioxus draft genome. Our expression survey showed that the maternal transcripts of a *Piwi* (*Bf-Piwil1*) and a Tudor family gene (*Bf-Tdrd7*) are colocalized with maternal *Vasa* in the germ plasm, suggesting that they might function in germ cell formation in amphioxus. To investigate the conservation of localized germ cell markers within cephalochordates further, we cloned *Vasa*, *Nanos*, *Piwi*, and *Tudor* cDNA fragments from other amphioxus species, including *B. belcheri* and *B. japonicum*, and analyzed their expression patterns. We also used immunostaining with Bf-Vasa antibody to trace the route taken by the putative PGCs to reach the gonad anlagen in developing amphioxus larvae.

## Methods

### Obtaining amphioxus adults and embryo collection

Gravid animals of the Florida amphioxus (*Branchiostoma floridae*) were collected in Tampa Bay, Florida, USA, during the summer breeding season. Gametes were obtained by electric stimulation. Fertilization and subsequent culturing of the embryos were carried out as previously described [22,23]. Two Asian amphioxus species, *Branchiostoma belcheri* and *Branchiostoma japonicum*, were collected from Kinmen Island near the Xiamen area in southeastern China. We cultured these two amphioxus species in our laboratory and obtained gametes and embryos during the breeding season using methods described previously [24]. Amphioxus embryos were staged according to Hirakow and Kajita [25,26], except that we further defined N0 (onset of neurulation, 0 somites, about 8.5 hours post-fertilization), N1 (early neurula, 1 to 3 somites, about 10 hours post-fertilization) and N2 (mid-neurula, 4 to 8 somites, about 15 hours post-fertilization) as described previously [27]. Our experimental research on amphioxus was approved by Academia Sinica Biosafety Review & Biomaterials and Lab Biosafety Information System (certificate number: BSF0412-00002656).

### Identification and cloning of *Piwi* and *Tudor* homologues in amphioxus and phylogenetic analyses

Amphioxus homologs of *Piwi/Argonaute* and *Tudor* family genes were identified from the *B. floridae* draft genome [28] using the *Basic Local Alignment Search Tool* (*BLAST*) with human and fly Piwi/Argonaute and Tudor proteins as queries. Identified gene models were subsequently used to search the amphioxus expressed sequence tag (EST) database and cDNA collection (*B. floridae* Gene Collection Release 1 [29]) to isolate the corresponding cDNA clones. Identified cDNA clones were sequenced from both ends by M13 forward and reverse primers as well as internal primers to obtain the complete nucleotide sequence of the inserts. For those genes whose corresponding cDNA could be identified from our cDNA collection, the translated amino acid sequences of the cDNA clones were used for protein domain analyses and phylogenetic analyses. Otherwise, the translated amino acid sequences of the predicted gene model deposited in the National Center for Biotechnology Information (NCBI) were used for subsequent analyses.

Multiple sequence alignments of PIWI and PAZ domains (for Piwi/Argonaute proteins) or Tudor domains (for Tudor domain-containing proteins) were generated by the Clustal X program [30] and optimized manually for phylogenetic reconstruction. The evolutionary distances were computed using the *p*-distance method [31] in units of the number of amino acid differences per site. Phylogenetic analysis was conducted with the MEGA5 program [32] using the neighbor-joining method [33] with 1,000 bootstrap replicates.

### Cloning of *B. belcheri* and *B. japonicum* cDNA

Unfertilized *B. belcheri* and *B. japonicum* eggs were collected, and total RNA was extracted using the Trizol reagent (Invitrogen) following the manufacturer’s instructions. RNA was further purified using the RNeasy Micro kit (Qiagen) and reverse-transcribed using the iScript cDNA synthesis kit (Bio-Rad), as described previously [34]. *B. belcheri* and *B. japonicum* cDNA encoding the *Vasa*, *Nanos*, *Piwil1*, and *Tdrd7* gene products was amplified by PCR using gene-specific primers designed from their *B. floridae* orthologs. The sequences of the primers are: Bb-Vasa, 5′-GTCATGCACCACAAGTCTCA-3′; 5′-CCAGTCTTCCTCGTCATCC-3′; Bj-Vasa, 5′-GTGAAGATGGCCATTTCTCAAGGGAGTGTC-3′; 5′-ACCACTTCCATTGTACTGTCTCCTGGTATC-3′; Bb-Nanos and Bj-Nanos, 5′-CGAGCGGTGTGTTTACGTTCTGGTAACGTC-3′; 5′-GGAAGTGACGTCTGAACGATACAGCCTGTC-3′; Bb-Piwil1 and Bj-Piwil1, 5′-GATGTCTTACGTGGAGTATTACGCC-3′; 5′-GGGTGTTGATTCGCTTCTTCAC-3′; Bb-Tdrd7, 5′-CTCAGTTGCCAAGTGGTATCAGTG-3′; 5′-CTGCTCCATAATCAGTGTTTGTCTCA-3′. Amplified cDNA fragments were cloned into the pGEM-T Easy vector (Promega).

### *In Situ* hybridization and immunostaining

For *B. floridae Piwi* and *Tudor* family genes, cDNA clones isolated from the cDNA collection were used to synthesize digoxigenin (DIG)-labeled anti-sense RNA probes for *in situ* hybridization. Template preparation, probe synthesis, and the procedure for single-color *in situ* hybridization were performed as described previously [11]. For double-fluorescent *in situ* hybridization (FISH), a fluorescein-labeled anti-sense *Bf-Vasa* RNA probe was used in combination with a DIG-labeled *Bf-Piwil1* or *Bf-Tdrd7* anti-sense RNA probes. A *Bf-Vasa* cDNA clone (bfne054i20) was used for probe synthesis, and the procedure for double-FISH was performed as described previously [11]. For *B. belcheri* and *B. japonicum* genes, cDNA fragments cloned in pGEM-T Easy vector were PCR-amplified by SP6 and T7 primers, and the PCR products were used for synthesizing DIG-labeled anti-sense RNA probes with SP6 or T7 RNA polymerase, depending on the orientation of the cDNA insert within the pGEM-T Easy vector. The *in situ* hybridization procedure for *B. belcheri* and *B. japonicum* embryos was the same as that for *B. floridae*.

Vasa immunostaining was performed using a polyclonal antibody against the *B. floridae* Vasa protein [11], and F-actin was stained with BODIPY FL Phallacidin (Invitrogen), as previously described [27]. For double-fluorescent staining of Vasa and F-actin, embryos were fixed in 4% PFA-MOPS-EGTA fixation buffer and then stored in PBS at 4°C until needed. In some cases, DAPI (Invitrogen, 1 mg/ml in PBS-Tween) and CellMask Orange (Invitrogen, 2.5 mg/ml in PBS-Tween) were used to mark the nuclei and general outline of the cells.

Images of *in situ* hybridization and immunostaining were taken using a Zeiss Axio Imager.A1 microscope with a Zeiss AxioCam HRc charge-coupled device (CCD) camera, or a Leica TCS-SP5-AOBS confocal microscope. Images of amphioxus adults were taken using a Leica Z16 APO microscope with a Leica DFC 300 FX CCD camera.

## Results and discussion

### Identification of *Piwi* subfamily genes in the amphioxus *B. floridae* and their expression patterns

The sequencing of the *B. floridae* genome and the release of the draft genome assembly by the Joint Genome Institute [28,35] allowed the identification of Piwi/Ago-related genes in amphioxus. Our initial translated nucleotide BLAST (TBLASTN) search and subsequent analyses of genome scaffolds identified seven gene models corresponding to the *Piwi/Ago* family genes (Additional file 1: Table S1). We found representative ESTs for five of these gene models from a *B. floridae* cDNA/EST database to support their expression. We noticed that within an 80 kb genomic region of scaffold 214, there were three gene models (protein/transcript ID: 126411, 126413, 91824) corresponding to the same cDNA cluster (Cluster ID: 02018, representative cDNA clone bfne050d12). It is probable that these three gene models were incorrectly annotated in the draft genome assembly and that they might instead constitute a single gene. Therefore, our search identified a total of five *Piwi/Ago* family members in the *B. floridae* genome.

**Figure 1:**
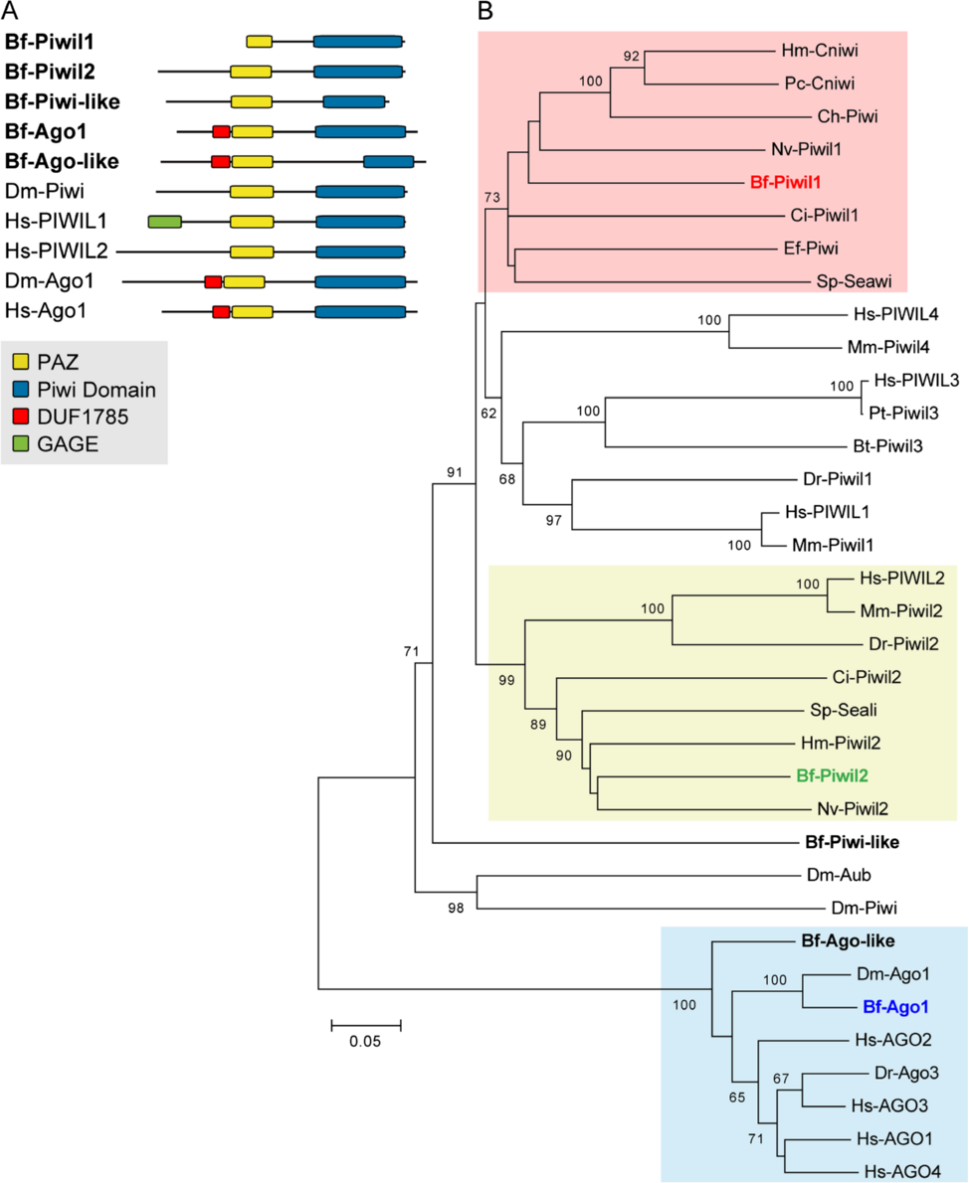
**Protein domain structure and phylogenetic analysis of *Branchiostoma floridae *Piwi/Ago family sequences.** (**A**) Schematic depiction of the composition of major domains in *B. floridae* Piwi and Argonaute (Ago) proteins (with abbreviation Bf), and related proteins from *Drosophila melanogaster* (Dm) and human (Hs). Conserved protein domain information is provided in Additional file 2: Table S2. (**B**) Phylogenetic tree of Piwi/Ago family proteins constructed using the neighbor-joining method. The percentage of replicate trees in which the associated taxa clustered together in the bootstrap test (1,000 replicates) is shown next to each branch. When the supporting percentage of a particular node is less than 60, the value is not shown on the node. The tree is drawn to scale, with branch lengths in the same units as those of the evolutionary distances used to infer the phylogenetic tree. The evolutionary distances were computed using the *p*-distance method and are presented as the number of amino acid differences per site. The analysis involved 35 amino acid sequences; their NCBI accession numbers are listed in Additional file 3. Species abbreviations: Bf, *Branchiostoma floridae* (Florida amphioxus); Bt, *Bos taurus* (cattle); Ch, *Clytia hemisphaerica* (hydra); Ci, *Ciona intestinalis* (tunicate); Dm, *Drosophila melanogaster* (fruit fly); Dr, *Danio rerio* (zebrafish); Ef, *Ephydatia fluviatilis* (sponge); Hm, *Hydra magnipapillata* (hydra); Hs, *Homo sapiens* (human); Mm, *Mus musculus* (mouse); Nv, *Nematostella vectensis* (sea anemone); Pc, *Podocoryne carnea* (jellyfish); Pt, *Pan troglodytes* (chimpanzee); Sp*, Strongylocentrotus purpuratus* (purple sea urchin).

The protein domain structures of these five amphioxus Piwi/Ago sequences and phylogenetic analysis revealed that three amphioxus sequences fell within the Piwi subfamily, and that the other two could be assigned to the Ago subfamily (Figure 1). Within the Piwi subfamily, we found that the sequence corresponding to the cDNA clone bfne050d12 (GenBank: KC516709) was grouped with cnidarian Piwi-like 1, *Ciona* Piwi-like 1, and sea urchin Seawi, so we tentatively named this sequence amphioxus Piwi-like 1 (Bf-Piwil1) (Figure 1B). The sequence corresponding to the cDNA clone bfga007o07 (GenBank: KC516710) was grouped with Piwi-like 2 sequences from other organisms, and we designated this sequence Piwi-like 2 (Bf-Piwil2). The third amphioxus Piwi sequence (protein ID 89999, GenBank accession number XP_002590851) fell at the base of most of the Piwi sequences (except for the *Drosophila* Piwi sequences) in our phylogenetic tree, suggesting that it might represent a more divergent Piwi member; we tentatively named this sequence amphioxus Piwi-like (Bf-Piwi-like). Regarding the two other amphioxus sequences that were grouped within the Ago subfamily (Figure 1B, blue shaded), the sequence corresponding to the cDNA clone bfga026e14 (GenBank: KC516711), which we named Ago1 (Bf-Ago1), was closely affiliated with *Drosophila* Ago1. The second amphioxus Ago sequence (protein ID 77832, accession number XP_002589390), which we tentatively named Ago-like (Bf-Ago-like), lay at the base of the Ago group and might be a divergent member of the Ago subfamily.

**Figure 2:**
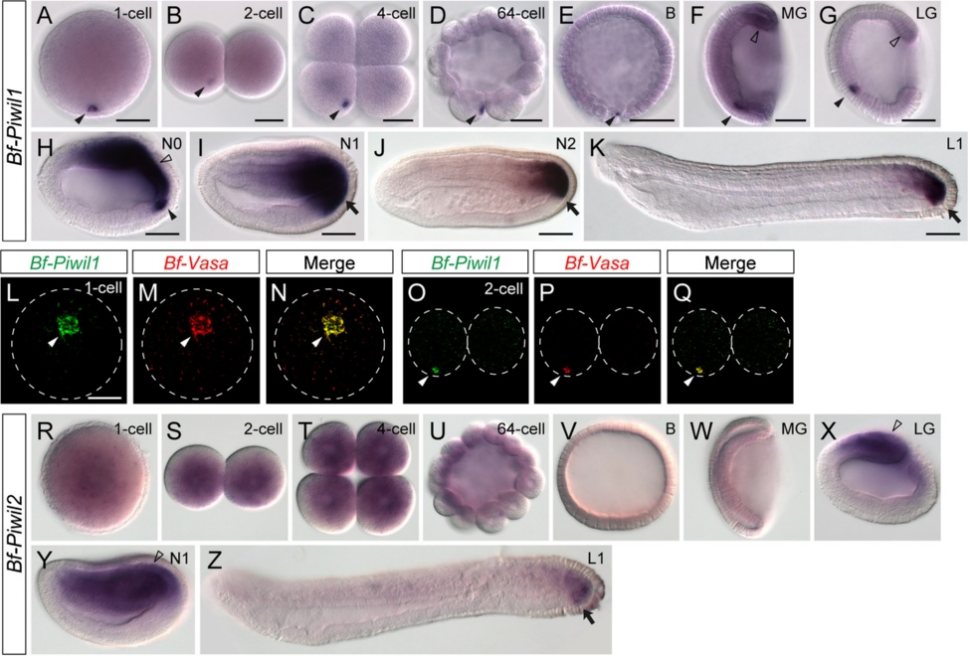
**Expression of *Bf-Piwil1 *and *Bf-Piwil2 *during embryonic and early larval development.** (**A**-**K**) *Bf-Piwil1* expression patterns. Embryos in **A**, **B**, **D**, **E** are in lateral view with the animal side up and vegetal side down; four-cell-stage embryo in **C** is in vegetal view; embryos and larvae in **F**-**K** are in left side view with the anterior to the left. Black arrowheads indicate aggregated maternal *Bf-Piwil1* mRNA, while hollow arrowheads indicate zygotic *Bf-Piwil1* expression around the blastopore during the gastrula stage. Black arrows indicate the expression of *Bf-Piwil1* in the putative PGCs located near the posterior growing zone. Scale bars, 50 μm, can be applied to embryos in the same developmental stage. (**L**-**Q**) Colocalization of *Bf-Piwil1* and *Bf-Vasa* mRNAs in one-cell- and two-cell-stage amphioxus embryos. **L**-**N** are in vegetal view, and O-Q are in lateral view. White arrowheads indicate *Bf-Piwil1* and *Bf-Vasa* signals. Dashed lines indicate the outline of the embryos. (**R**-**Z**) *Bf-Piwil2* expression patterns. Embryos in **R**, **S**, **U**, **V** are in lateral view with the animal side up and vegetal side down; four-cell-stage embryo in **T** is in vegetal view; embryos and larvae in **W**-**Z** are in left side view with the anterior to the left. Hollow arrowheads indicate the zygotic *Bf-Piwil2* expression in the dorsal side of the late gastrula and early-neurula-stage embryos. Arrow indicates the expression of *Bf-Piwil2* in the posterior part of the larva. Stage abbreviations: B, blastula, 4 hpf; MG, mid-gastrula, 6 hpf; LG, late gastrula, 7 hpf; N0, onset of neurulation, 8.5 hpf; N1, early neurula, 10 hpf; N2, mid-neurula, 15 hpf; L1, early larva, 24 hpf.

We have identified ESTs and cDNA clones derived from three *Piwi/Ago* family genes, namely *Bf-Piwil1*, *Bf-Piwil2*, and *Bf-Ago1*; however, we did not find any EST evidence for *Bf-Piwi-like* and *Bf-Ago-like* to support their expression. To investigate whether *Bf-Piwil1*, *Bf-Piwil2* function in amphioxus PGC formation, we used their cDNA clones to synthesize anti-sense riboprobes to examine their developmental expression. Whole-mount *in situ* hybridization demonstrated clear maternal *Bf-Piwil1* transcripts within early amphioxus embryos (Figure 2A-E). Like amphioxus *Vasa* and *Nanos* transcripts [11], maternal *Bf-Piwil1* transcripts asymmetrically aggregated as a compact granule near the vegetal pole in fertilized eggs (Figure 2A; black arrowhead), and some diffused *Bf-Piwil1* transcripts could also be detected throughout the entire embryo. During the subsequent cleavages, the aggregated maternal *Bf-Piwil1* transcripts were inherited by only one of the blastomeres until the late blastula or early gastrula stage (Figure 2B-E; black arrowheads). To confirm that maternal *Bf-Piwil1* aggregated in the vegetal pole plasm [11,14], which is the putative germ plasm in amphioxus, we performed double-FISH using *Bf-Piwil1* and *Bf-Vasa* probes. Maternal *Bf-Piwil1* transcripts were indeed colocalized with transcripts of germline marker *Bf-Vasa* during early embryogenesis (Figure 2L-Q), suggesting that *Bf-Piwil1* is a component of the germ plasm in amphioxus. This observation is consistent with the notion that *Piwi* homologs play important roles in germ cell development in other organisms [4]. During gastrulation, the vegetal plate invaginates to form the future mesendoderm, and we found the maternal *Bf-Piwil1-*containing cell was located in the ventral mesendoderm and started to divide into multiple cells (Figure 2F, G; black arrowheads). These cells are the putative PGCs in amphioxus [11]. From the mid-gastrula stage, *Bf-Piwil1* was also expressed zygotically around the blastopore (Figure 2F, G, hollow arrowheads). During the neurula stage, the zygotic *Bf-Piwil1* expression was upregulated in the posterior mesendoderm near the closing blastopore, and the putative PGCs appeared to move toward the posterior end to connect with this zygotic *Bf-Piwil1* expression domain in the posterior ventral endoderm (Figure 2H-J; black arrowhead and arrows). In the early larval stage, *Bf-Piwil1* was expressed in the posterior tail bud region, which is considered the growth zone for body elongation [36], and in the nascent somites (Figure 2K). In summary, *Bf-Piwil1* is expressed in an identical pattern to *Bf-Vasa* and *Bf-Nanos*[11] in the putative PGCs and in the undifferentiated somatic stem cells in amphioxus, suggesting that it might play roles in both early PGC determination and subsequent somatic stem cell development. This is consistent with recent observations that a core set of genetic toolkits play dual roles in germline stem cells and somatic stem cells in diverse metazoan animals [3,37,38].

In contrast, although we also detected maternal transcripts of *Bf-Piwil2* in early amphioxus embryos, we did not observe highly localized *Bf-Piwil2* transcripts in the germ plasm. *Bf-Piwil2* transcripts were distributed uniformly in the one-cell-stage and cleavage-stage embryos (Figure 2R-U). This observation suggests that *Bf-Piwil2* might have different functions from that of *Bf-Piwil1* during early embryogenesis. From the blastula stage, the maternal *Bf-Piwil2* transcript was no longer detectable, and no zygotic expression could be detected from the blastula to the mid-gastrula stage (Figure 2V, W). Later in the late-gastrula and early-neurula stages, *Bf-Piwil2* began to be expressed in the dorsal mesendoderm (Figure 2X, Y), and subsequently its expression became more restricted to the posterior tail bud region (Figure 2Z), suggesting that it might also function in the larval growth zone of amphioxus.

Unfortunately, we could not detect the spatial expression pattern of *Bf-Ago1* by *in situ* hybridization using the cDNA clone identified in the current study. We suspect that this was due to the low expression level of this gene during amphioxus development. This possibility is supported by the EST count data from our cDNA/EST database [29]. Because the cDNA libraries for this EST project were not normalized, presumably the EST count of a particular cDNA cluster could be used as rough ‘digital Northern’ data representing its expression level [29,39]. We identified only one EST clone for *Bf-Ago1* (cDNA cluster 17469) from the EST count data, which was much less than the other genes, such as *Bf-Piwil1* (cDNA cluster 02018: 17 counts in total) or *Bf-Vasa* (cDNA cluster 00124: 27 counts in total), whose expression was easily detected by *in situ* hybridization. Therefore, more sensitive methods for detecting gene expression or improvement of our *in situ* hybridization protocols might be needed to study the expression pattern of *Bf-Ago1* further.

### Identification of genes encoding Tudor domain proteins in *B. floridae* and the localization of *Tdrd7* maternal transcripts in the putative PGC

**Figure 3:**
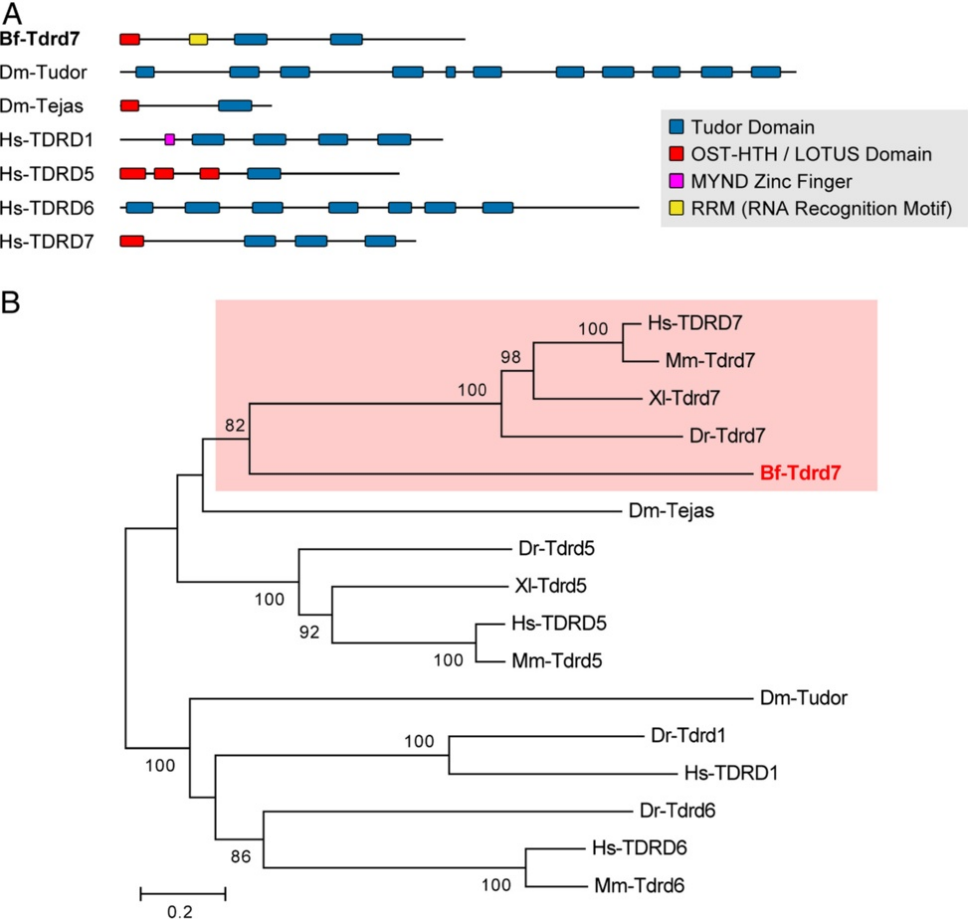
**Protein domain structure and phylogenetic analysis of Tudor domain-containing 7 sequences.** (**A**) Schematic depiction of the composition of major domains in *B. floridae* Tudor domain-containing 7 (Tdrd7) protein (predicted from clone bfga025n02) and related proteins from *Drosophila melanogaster* (Dm) and human (Hs). Conserved protein domain information is provided in Additional file 2: Table S2. (**B**) Phylogenetic tree of *B. floridae* Tdrd7 and other related proteins constructed using the neighbor-joining method. The percentage of replicate trees in which the associated taxa clustered together in the bootstrap test (1,000 replicates) is shown next to each branch. When the supporting percentage of a particular node is less than 60, the value is not shown on the node. The tree is drawn to scale, with branch lengths in the same units as those of the evolutionary distances used to infer the phylogenetic tree. The evolutionary distances were computed using the *p*-distance method and are presented as the number of amino acid differences per site. The analysis involved 16 amino acid sequences, and their NCBI accession numbers are listed in Additional file 3. Species abbreviations: Bf, *Branchiostoma floridae* (Florida amphioxus); Dm, *Drosophila melanogaster* (fruit fly); Dr, *Danio rerio* (zebrafish); Hs, *Homo sapiens* (human); Mm, *Mus musculus* (mouse); Xl, *Xenopus laevis* (African clawed frog).

We searched the assembled *B. floridae* draft genome using *Drosophila* and human Tudor homologs as queries and identified 15 gene models that encoded proteins containing Tudor domains (Additional file 4: Table S3; Additional file 5: Figure S1). Among these identified gene models, ten had corresponding ESTs in the database to support their expression. After sequencing these cDNA clones, we noted that many of the identified amphioxus cDNA clones only contained the partial open reading frames (ORFs) of the Tudor domain-containing proteins, which impeded our ability to construct a robust phylogenetic tree for every amphioxus Tudor domain-containing sequence. Therefore, we focused on one gene model (protein/transcript ID: 125731), whose corresponding cDNA had the complete ORF for a Tudor homolog. The cDNA clone (bfga025n02, Cluster ID 17456) of this gene was 5,120 bp in length, with a putative ORF encoding a protein of 1,279 amino acids (GenBank: KC516712). Our analysis indicated that in addition to having two Tudor motifs, this protein also had a RRM RNA recognition domain and a recently named LOTUS domain [40] near its N-terminus (Figure 3A). Our phylogenetic analysis showed that this sequence was closely grouped with Tudor domain-containing 7 (Tdrd7) homologs from vertebrates (Figure 3B). Therefore, we named this gene Tudor domain-containing 7 (Bf-Tdrd7). Bf-Tdrd7 plus other Tdrd7 homologs were closely related to *Drosophila* Tejas and vertebrate Tdrd5 homologs, all of which have the LOTUS domain (Figure 3B), suggesting that they may constitute a distinct subfamily of Tudor domain-containing proteins.

**Figure 4:**
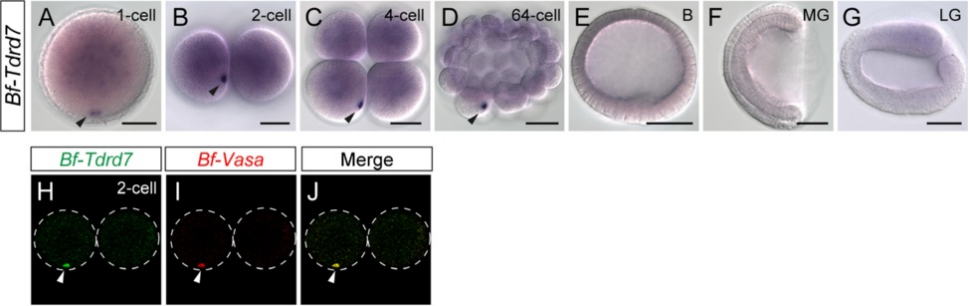
**Expression of *Bf-Tdrd7 *during embryonic development.** (**A**-**G**) *Bf-Tdrd7* transcripts are only expressed maternally until late cleavage stage, and no zygotic *Bf-Tdrd7* expression can be detected. Embryos in **A**, **D**, **E** are in lateral view with the animal side up and vegetal side down; **B** and **C** are in vegetal view; embryos in **F** and **G** are in left view with the anterior to the left. Black arrowheads indicate the aggregated maternal *Bf-Tdrd7* mRNA. Scale bars, 50 μm, can be applied to embryos in the same developmental stage. (**H**-**J**) Colocalization of *Bf-Tdrd7* and *Bf-Vasa* mRNAs in cleavage-stage amphioxus embryo. Images are in lateral view. White arrowheads indicate *Bf-Tdrd7* and *Bf-Vasa* signals. Dashed lines indicate the outline of the embryos.

Vertebrate *Tdrd5*, *Tdrd7*, and *Drosophila tejas* are expressed in the germline lineage and are essential for germ cell function [41-43]. To investigate whether *Bf-Tdrd7* is also localized in amphioxus germ plasm, we used *in situ* hybridization to examine its expression pattern. Maternal *Tdrd7* transcripts were present in one-cell-stage amphioxus embryos, and the transcripts were more highly aggregated in one spot near the vegetal pole (Figure 4A; black arrowhead). During subsequent cleavages, this spot remained visible in one blastomere (Figure 4B-D; black arrowheads). We also performed double-FISH using *Bf-Tdrd7* and *Bf-Vasa* probes, which confirmed that *Bf-Tdrd7* and *Bf-Vasa* were indeed colocalized in the germ plasm (Figure 4H-J), suggesting that *Bf-Tdrd7* is probably also involved in PGC formation in amphioxus. Interestingly, after the blastula stage, this *Bf-Tdrd7* signal disappeared, and no clear zygotic expression of *Bf-Tdrd7* could be detected by *in situ* hybridization (Figure 4E-G). This expression pattern is in clear contrast with the other three germline markers (*Bf-Vasa*, *Bf-Nanos*, and *Bf-Piwil1*), which are expressed zygotically after the blastula stage not only in the forming PGCs but also in the posterior tail bud region (Figure 2F-K, and [11]). *Bf-Tdrd7* is only expressed maternally in the germ plasm, suggesting that it might function exclusively in the early steps of germ cell specification in amphioxus. Therefore, *Bf-Tdrd7* could represent one of the molecular components that distinguish between the amphioxus germline stem cells and somatic stem cells.

### The expression of germ cell-related genes in *B. belcheri* and *B. japonicum* demonstrates the overall conservation of the PGC specification mechanism among *Branchiostoma* amphioxus species

We have demonstrated that Florida amphioxus *B. floridae* has maternally localized germ plasm that contains the commonly recognized germline markers *Vasa*, *Nanos*, *Piwi*, and *Tudor*. This suggests that this species may use the preformation mechanism for PGC specification. To test the conservation of the preformation mechanism for PGC specification in cephalochordates, we investigated the expression pattern of these common germline markers in two distantly related Asian amphioxus species, *B. belcheri* and *B. japonicum*. Based on mitochondria DNA data, these two amphioxus species native to the West Pacific Ocean form a distinct phylogenetic group and are estimated to have diverged from the Atlantic amphioxus group (*B. floridae* and *B. lanceolatum*) about 150 million years ago [44]. Using PCR with gene-specific primers, we amplified and cloned the cDNA fragments of *Vasa*, *Nanos*, and *Piwil1* homologs from both *B. belcheri* and *B. japonicum*; we also obtained *Tdrd7* cDNA from *B. belcheri* (GenBank: KC516713-KC516719), but not from *B. japonicum*. We used these cDNA fragments for the expression survey described.

**Figure 5:**
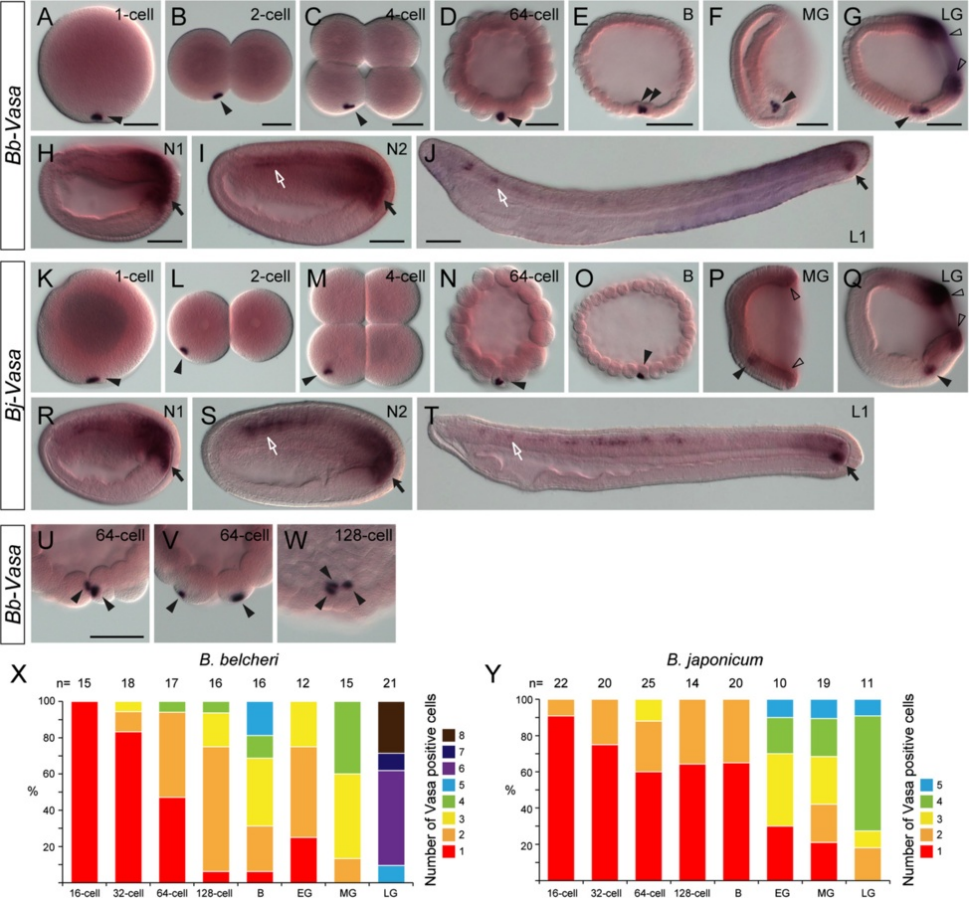
**Expression patterns of *Vasa *homologs in two Asian amphioxus species (*B. belcheri *and *B. japonicum*) are mostly identical to that of *B. floridae*.** (**A**-**T**) *Bb-Vasa* and *Bj-Vasa* expression patterns. Embryos in **A**, **B**, **D**, **E**, **K**, **L**, **N**, and **O** are in lateral view with the animal side up and vegetal side down; four-cell stage embryos in **C** and **M** are in vegetal view; embryos and larvae in **F**-**J** and **P**-**T** are in left side view with the anterior to the left. Black arrowheads indicate aggregated maternal *Vasa* mRNA, while hollow arrowheads indicate zygotic *Vasa* expression around the blastopore during the gastrula stage. Black arrows indicate expression in the putative PGCs located near the posterior growing zone. White arrows indicate the weak expression of *Vasa* in the anterior somites. Scale bars, 50 μm. (**U**-**W**) Higher-magnification images showing that aggregated maternal *Bb-Vasa* transcripts are frequently observed in multiple blastomeres in late-cleavage-stage *B. belcheri* embryos. Arrowheads indicate the individual *Bb-Vasa* mRNA granules in different blastomeres. Scale bars, 50 μm, can be applied to embryos in the same developmental stage. (**X**-**Y**) Distribution of the observed cell numbers harboring the maternal *Vasa* mRNA granules during *B. belcheri* and *B. japonicum* early embryogenesis. The sample size for each developmental stage is shown on top of the graphs.

In *B. belcheri* and *B. japonicum*, we observed that *Vasa* transcripts were also present maternally and localized asymmetrically to one spot near the vegetal cortex in fertilized eggs (Figure 5A, K). During the first few cleavages, this compact *Vasa* spot was still present in only one of the blastomeres (Figure 5B-D, L-N). This distribution pattern is identical to that in *B. floridae*[11]. Interestingly, we noted that in *B. belcheri*, the compacted *Vasa* transcripts appeared to be split and distributed into multiple blastomeres earlier than those in *B. japonicum* (Figure 5D-F, N-P) and *B. floridae*[11]. From the 64-cell stage, more than 50% of the *B. belcheri* embryos had multiple cells that harbored *Vasa* transcripts (Figure 5U-W, X), and the transcripts were still tightly aggregated with the cell cortex within each cell (Figure 5U-W). By the blastula stage, over 90% of the *B. belcheri* embryos had multiple *Vasa* transcript-containing cells. In *B. japonicum* and *B. floridae*, the compacted *Vasa* transcripts usually remained in a single blastomere until the blastula stage, and more frequently the *Vasa* transcripts began to appear in multiple cells in the early or mid-gastrula stage (Figure 5O, P, Y).

During gastrulation, the expression of *Vasa* was mostly identical among the different *Branchiostoma* amphioxus species. The cells harboring the *Vasa* transcripts could be identified as one cluster of cells located in the ventral mesendoderm (Figure 5F, G, P, Q; black arrowheads). *Bb-Vasa* and *Bj-Vasa* were also expressed zygotically around the blastopore during the gastrula stage (Figure 5G, P, Q; hollow arrowheads). Subsequently, the ventral *Vasa*-positive cell cluster appeared to move posteriorly during the neurula stage, and it eventually associated with the posterior tail bud, which also expressed *Vasa* zygotically (Figure 5H, I, R, S; black arrows). In neurula-stage embryos, *Bb-Vasa* and *Bj-Vasa* were also expressed weakly in the somites (Figure 5I, S; white arrows). This expression domain was not described clearly in our previous study using *B. floridae* embryos [11], and after careful re-examination we confirmed that *Bf-Vasa* was indeed also expressed weakly in the somites (see Figure 4N in [11]). In the larval stage, clear *Bb-Vasa* and *Bj-Vasa* expression was present near the tail bud (Figure 5J, T; black arrows), as with *Bf-Vasa*; some weak expression was also detectable in somites at this stage (Figure 5J, T; white arrows).

**Figure 6:**
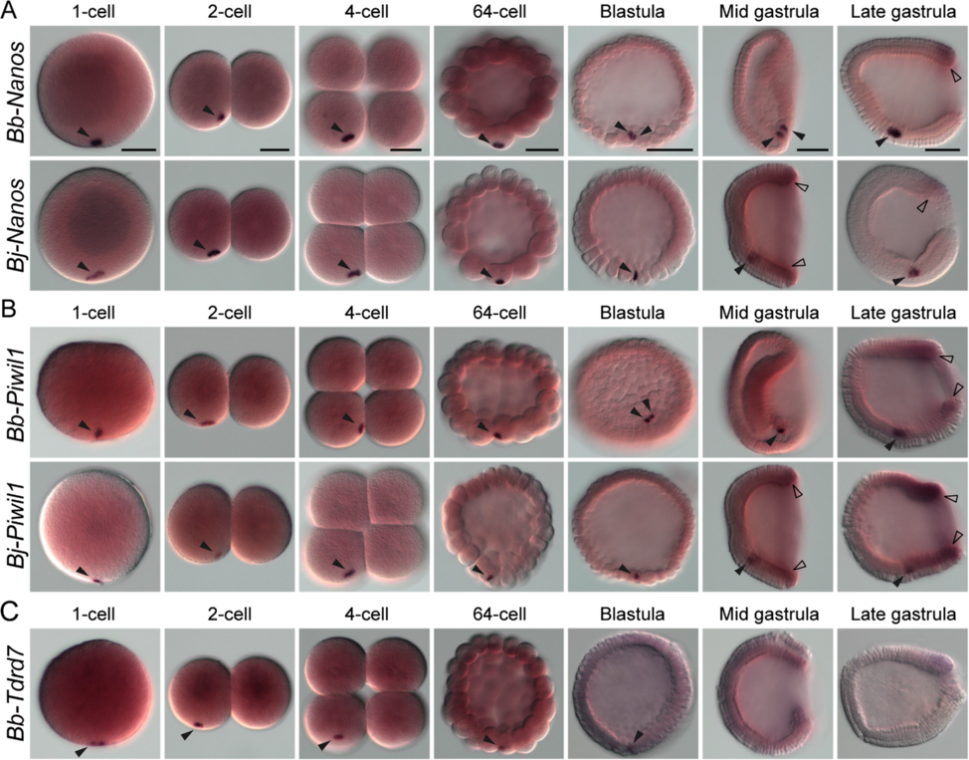
**Expression patterns of *Nanos*, *Piwil1*, and *Tdrd7 *homologs in *B. belcheri *and *B. japonicum *during embryonic development.** (**A**) *Bb-Nanos* and *Bj-Nanos* expression patterns. (**B**) *Bb-Piwil1* and *Bj-Piwil1* expression patterns. (**C**) *Bb-Tdrd7* expression patterns. One-cell-, two-cell-, 64-cell-, and blastula-stage embryos are in lateral view with the animal side up and vegetal side down; four-cell-stage embryos are in vegetal view; mid-gastrula and late-gastrula-stage embryos are in left side view with the anterior to the left. Black arrowheads indicate the aggregated maternal mRNA, while hollow arrowheads indicate the zygotic expression around the blastopore during the gastrula stage. Scale bars, 50 μm, can be applied to embryos in the same developmental stage.

The maternal transcripts of *Nanos* and *Piwil1* homologs were also localized asymmetrically in vegetal germ plasm in both *B. belcheri* and *B. japonicum* during early embryogenesis (Figure 6; black arrowheads)*.* After the gastrula stage, these genes were also expressed zygotically around the blastopore (Figure 6; hollow arrowheads) and eventually expressed in the tail bud region (Additional file 6: Figure S2). The expression patterns of these two genes were almost identical to that of *Vasa* in each respective species (Figures 5, 6, Additional file 6: Figure S2), suggesting that they are coexpressed with *Vasa* in all the amphioxus species that we studied. Like *Bf-Tdrd7*, *Bb-Tdrd7* was only expressed maternally and localized in the vegetal germ plasm until the early blastula stage in *B. belcheri* (Figure 6C), and no *Bb-Tdrd7* transcript was detected in later developmental stages. In summary, our expression survey reveals a general conservation of localized maternal transcripts of these common germline markers during early embryogenesis among different amphioxus species.

### Distribution of Vasa-positive cells in amphioxus larvae and the movement of these cells to the gonad anlagen during development

**Figure 7:**
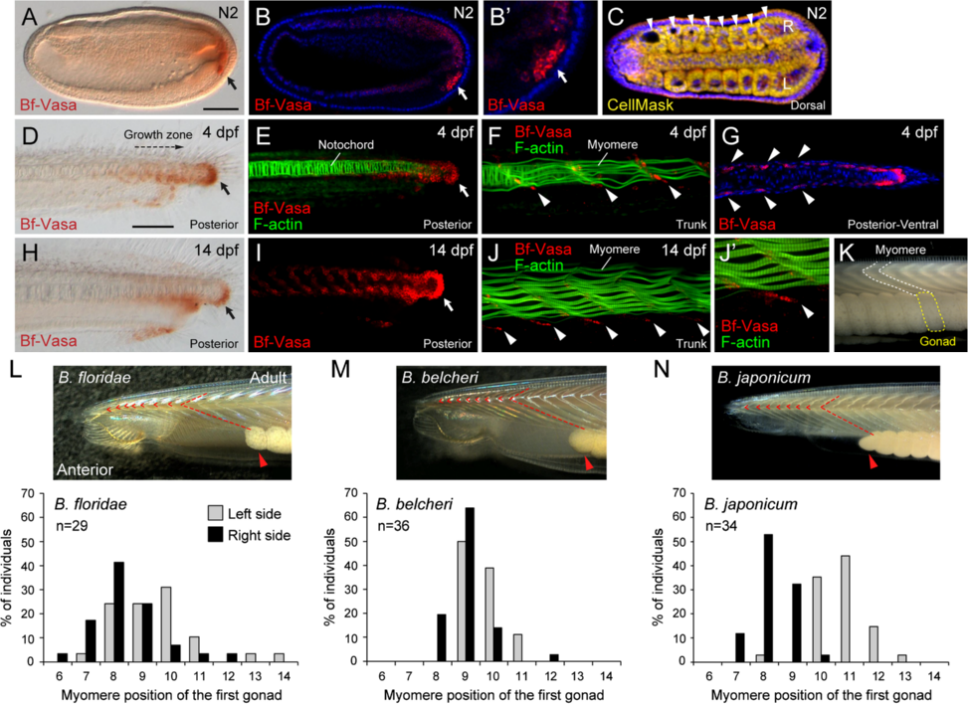
**Germ cell labeling with Vasa antibody (Bf-Vasa) during embryogenesis and larval development in *B. floridae*, and position of mature gonads in three amphioxus species.** (**A**) Bf-Vasa antibody staining showing putative PGCs (arrow, brown signal) at ventral tail bud at N2 neurula stage. Scale bar, 50 μm. (**B**) Fluorescent immunostaining using Bf-Vasa antibody showing putative PGCs (arrow, red signal) at stage N2. (**B**′) Higher-magnification image of posterior end of embryo in B, showing putative PGCs. (**C**) Dorsal view of embryo in **B** with CellMask counterstaining to show numbers of somites (arrowheads); R, right; L, left. (**D**-**G**) PGC staining in 4 days post-fertilization (dpf) larva. Arrows in **D, E** indicate Vasa-positive cells located near the tail bud. Arrowheads in **F**, **G** indicate Vasa-positive cells distributed along myomere boundaries. **G** Ventral view showing distribution of Vasa-positive cells on both sides of body in *B. floridae* larva. (**H**-**J**) PGC staining in 14 dpf larva. Arrows in **H**, **I** indicate Vasa-positive cells located near the tail bud. Arrowheads in J indicate Vasa-positive cells concentrated at the ventral extremity of the myomeres. (**J′**) Higher-magnification image of ventral tip of myomere, showing putative PGCs. (**K**) Lateral view of adult amphioxus showing gonad morphology. White dashed line indicates outline of a myomere, while yellow dashed line encircles gonad underneath that particular myomere. (**L**-**N**) Position of most anterior gonad (in terms of myomere level) in *B. floridae*, *B. belcheri*, and *B. japonicum*. Photos are left side view of adult amphioxus with the anterior to the left. Red arrowhead indicates first gonad; red dashed line indicates the myomere associated with first gonad; red chevrons indicate anterior gonad-less myomeres. Bar graphs show distribution of myomere positions of first gonad (the most anterior) in three amphioxus species. Sample sizes are indicated under species names.

We have shown that during the neurula stage, the amphioxus PGCs, presumably specified by the maternal germline markers, move toward the posterior end and join with the tail bud cells, the putative multipotent somatic stem cells that also express *Vasa*, *Nanos*, and *Piwi* homologs ([11] and this study). To investigate how these amphioxus PGCs reach their final destination in the gonad anlagen located in the most ventral part of the somites, we used immunostaining with Bf-Vasa antibody to trace the PGCs in later development. As described previously [11], we observed that the presumed PGCs usually arrived at the ventral tail bud during the N2 stage (Figure 7A-C), when the embryos were developing their fifth to eighth pairs of somites. These presumed PGCs were recognized by their larger nuclei and stronger Vasa signals (Figure 7B, B′; white arrow) compared with the tail bud cells and the newly forming somites, which showed weaker Vasa signals. We suspect that these Vasa-positive cells in the tail bud and somites are the somatic stem cells. With the continuous budding of the posterior somites from the tail bud in amphioxus larvae, we observed that the strongest Vasa signal was in the tail bud region near the growth zone (Figure 7D, E). Moreover, in the middle and posterior parts of the larvae, we observed clear Vasa-positive cells distributed along the boundaries between the developing muscle segments (myomeres) (Figure 7F, G). In premetamorphosis larvae (14 days post-fertilization), the posterior tail bud maintained a strong Vasa signal (Figure 7H, I); in the trunk region, the Vasa-positive cells became restricted to the most ventral space of the V-shaped myomeres (Figure 7J, J′). This distribution pattern is consistent with previous histological descriptions of the earliest recognizable PGCs in amphioxus larvae located in the ventral myoseptal walls of the myomeres [13], and the pattern corresponds to the future position of the gonads (Figure 7K), suggesting that these Vasa-positive cells represent the PGCs that have settled into the gonad anlagen. We could observe more than eight Vasa-positive cells in this position in 14-day-old amphioxus larvae (Figure 7J′); however, we were not able to determine the exact number of putative PGCs that settle into each gonad anlagen, because we observed quite variable numbers of Vasa-positive cells in this position among different individuals, or even among different gonad anlagen sites within one individual. Further study will be required to confirm this number.

From these observations, we hypothesize that after the N2 stage, when the putative PGCs join the posterior tail bud, these amphioxus PGCs proliferate with the tail bud cells during the posterior elongation. With the addition of each new posterior somite, some PGCs are partitioned and deposited near the forming myomere boundaries. This hypothesis is consistent with the unique somitogenesis process in amphioxus and the position of the gonads in its body. The first few amphioxus somites are formed by a pouching (enterocoelic) process of the paraxial mesoderm during the N1 and early N2 stages; conversely, the posterior amphioxus somites are derived by a splitting (schizocoely) process from the tail bud after the N2 stage [36,45]. One can imagine that if our hypothesis is correct, the amphioxus PGCs must be absent from the anterior somites (which are formed by an enterocoelic process in the N1 stage) and must be present only in the posterior somites (which are derived from the tail bud). To test this, we carefully determined the position of the first pair of gonads (on the left and right sides of the body, respectively) and their associated myomeres in all three amphioxus species. We found that the most anterior gonad always registered with the sixth to eighth myomere in all three *Branchiostoma* amphioxus species that we examined (Figure 7L-N), and no gonads were observed under the first five myomeres. We noted that the right and left gonads were staggered and out of register, with one or two gonads on the right side appearing further ahead than those on the left (Figure 7L-N, bar graphs). This pattern appears to be closely associated with the asymmetric budding of the left and right somites during embryogenesis [36]. Currently, we cannot completely exclude the possibility that some putative PGCs near the tail bud would not only partition into somite boundaries during somitogenesis, but also could migrate across boundaries to populate more anterior somites. More detailed observations with specific cell labeling techniques are required to answer this question.

During the morphogenesis of the V-shaped myomeres, the PGCs became restricted to the ventral tip of the myoseptal walls (myosepta) to form the gonad anlagen (Figure 7F, J, J′). There are two possible scenarios to explain this ventral localization of PGCs along the myosepta during myomere morphogenesis. First, the Vasa-positive cells detected along the dorso-ventral axis of the myomere boundaries might initially be composed of both somatic stem cells and PGCs. Judging from their original positions in the tail bud area, it is possible that the dorsal Vasa-positive cells are the somatic stem cells, and they could eventually cease to express Vasa during later larval development; only the Vasa-positive cells located near the ventral myoseptum tips represent the *bona fide* PGCs and continue to express Vasa. Alternatively, the putative PGCs might be initially scattered along the myomere boundaries, and later move ventrally to populate the ventral myoseptum tips during myomere maturation. We are not yet able to differentiate between these two possibilities from our data. Further study is required to resolve this issue.

## Conclusions

In this study, we show that in three different *Branchiostoma* amphioxus species, putative maternal germ plasm components of *Vasa*, *Nanos*, *Piwi*, and *Tudor* transcripts are asymmetrically localized in a compact granule near the vegetal cortex of the fertilized eggs and are subsequently inherited by the putative PGCs. Therefore, we have presented additional evidence to support the general conservation of the preformation mode of PGC specification in *Branchiostoma* amphioxus. Besides *Branchiostoma* species, which have paired gonads, there is another group of amphioxus, including the *Epigonichthys* and *Asymmetron*, that have gonads only on the right side of the body [13,46,47]. Little is known about the biology of *Epigonichthys* and *Asymmetron* amphioxus, and only recently did researchers successfully obtain laboratory spawning from an *Asymmetron* amphioxus (*Asymmetron lucayanum*) and describe its early development [46,47]. Thus, it would be necessary to extend this survey of germ plasm components to the more distantly related group of amphioxus, such as the *Asymmetron* species, to confirm the generality of the currently described germ cell specification mechanism in cephalochordates. Furthermore, using Vasa antibody staining to trace the distribution of PGCs in *Asymmetron* species during late embryogenesis (N2 to larval stage) might help to explain how gonads can form only on the right side of its body.

## Abbreviations

Ago: Argonaute; BLAST: Basic local alignment search tool; CCD: Charge-coupled device; dpf: Days post-fertilization; DIG: Digoxigenin; EST: Expressed sequence tag; FISH: Fluorescent *in situ* hybridization; hpf: Hours post-fertilization; NCBI: National Center for Biotechnology Information; ORF: Open reading frames; PBS: Phosphate-buffered saline; PCR: Polymerase chain reaction; PGC: Primordial germ cell; TBLASTN: Translated nucleotide BLAST; Tdrd: Tudor domain.

## Competing interests

The authors declare that they have no competing interests.

## Authors’ contributions

QJZ carried out molecular cloning, *in situ* hybridization, immunohistochemistry, microscopic imaging, and data analysis. YJL carried out gene orthology and phylogenetic analyses, *in situ* hybridization, immunohistochemistry, microscopic imaging, and data analysis, and prepared the figures. HRW carried out *in situ* hybridization, immunohistochemistry, and microscopic imaging. YTC carried out molecular cloning, *in situ* hybridization, and microscopic imaging. JKY conceived of the study, designed the study, contributed to imaging and analysis of the data, and wrote the manuscript. All authors read and approved the final manuscript.

## Supplementary Material

Additional file 1: Table S1 Piwi/Ago family genes identified in the *B. floridae* draft genome. Click here for file

Additional file 2: Table S2 Conserved protein domain information for Piwi/Ago family and Tudor domain family proteins. Click here for file

Additional file 3 NCBI accession numbers for the sequences used in the phylogenetic analyses. Click here for file

Additional file 4: Table S3 Tudor domain family genes identified in the *B. floridae* draft genome. Click here for file

Additional file 5: Figure S1 Schematic depiction of the composition of major domains in *B. floridae* Tudor domain-containing proteins. Click here for file

Additional file 6: Figure S2 Expression patterns of *Nanos* and *Piwil1* homologs in *B. belcheri* and *B. japonicum* during the neurula and larval stages. Black arrowhead indicates the expression in the putative PGCs, and hollow arrowhead indicates the zygotic expression around the tail bud region. Arrows indicate the merged expression domain in the putative PGCs and the posterior tail bud. Click here for file
