# Supplementary material for: Expression of germline markers in three species of amphioxus supports a preformation mechanism of germ cell development in cephalochordates
Source: EvoDevo. 2013 Jun 18;4:17. doi: 10.1186/2041-9139-4-17 (PMC3735472; doi:10.1186/2041-9139-4-17)
Supplement: Additional file 3 — NCBI accession numbers for the sequences used in the phylogenetic analyses. [file 2041-9139-4-17-S3.pdf]

**Additional file 3: NCBI accession numbers for the sequences used in the phylogenetic analyses**

1) NCBI accession numbers used for Piwi phylogenetic tree in Figure 1B:

Bt-Piwil3 (XP_872390); Ch-Piwi (ABY67112); Ci-Piwil1 (XP_002120252); Ci-Piwil2 (XP_002130490); Dm-Ago1 (AAF58314); Dm-Aub (AAF53046); Dm-Piwi (NP_476875); Dr-Ago3 (NP_001153500); Dr-Piwil1 (NP_899181); Dr-Piwil2 (NP_001073668); Ef-Piwi (BAJ07610); Hm-Piwil1 (XP_002155913); Hm-Piwil2 (XP_002162153); Hs-AGO1 (NP_036331); Hs-AGO2 (NP_036286); Hs-AGO3 (NP_079128); Hs-AGO4 (NP_060099); Hs-PIWIL1 (NP_004755); Hs-PIWIL2 (NP_001129193); Hs-PIWIL3 (NP_001008496); Hs-PIWIL4 (NP_689644); Mm-Piwil1 (NP_067286); Mm-Piwil2 (BAA93706); Mm-Piwil4 (NP_808573); Nv-Piwil1 (XP_001641994); Nv-Piwil2 (XP_001626127); Pc-Cniwi (AAS01181); Pt-Piwil3 (XP_003317193); Sp-Seali (NP_001107667); Sp-Seawi (NP_999765).

2) NCBI accession numbers used for Tudor phylogenetic tree in Figure 3B:

Dm-Tejas (AAF58263); Dm-Tudor (AAF46693); Dr-Tdrd1 (NP_001157500); Dr-Tdrd5 (AAI34986); Dr-Tdrd6 (NP_001182210); Dr-Tdrd7 (ABR24798); Hs-Tdrd1 (AAH35010); Hs-Tdrd5 (AAI30533); Hs-Tdrd6 (EAX04300); Hs-TDRD7 (NP_055105); Mm-Tdrd5 (AAH99972); Mm-Tdrd6 (AAI45249); Mm-Tdrd7 (CAM17034); Xl-Tdrd5 (NP_001090599); Xl-Tdrd7 (NP_001084569).
