# Supplementary figures and images for: Expression of germline markers in three species of amphioxus supports a preformation mechanism of germ cell development in cephalochordates
Source: EvoDevo. 2013 Jun 18;4:17. doi: 10.1186/2041-9139-4-17 (PMC3735472; doi:10.1186/2041-9139-4-17)

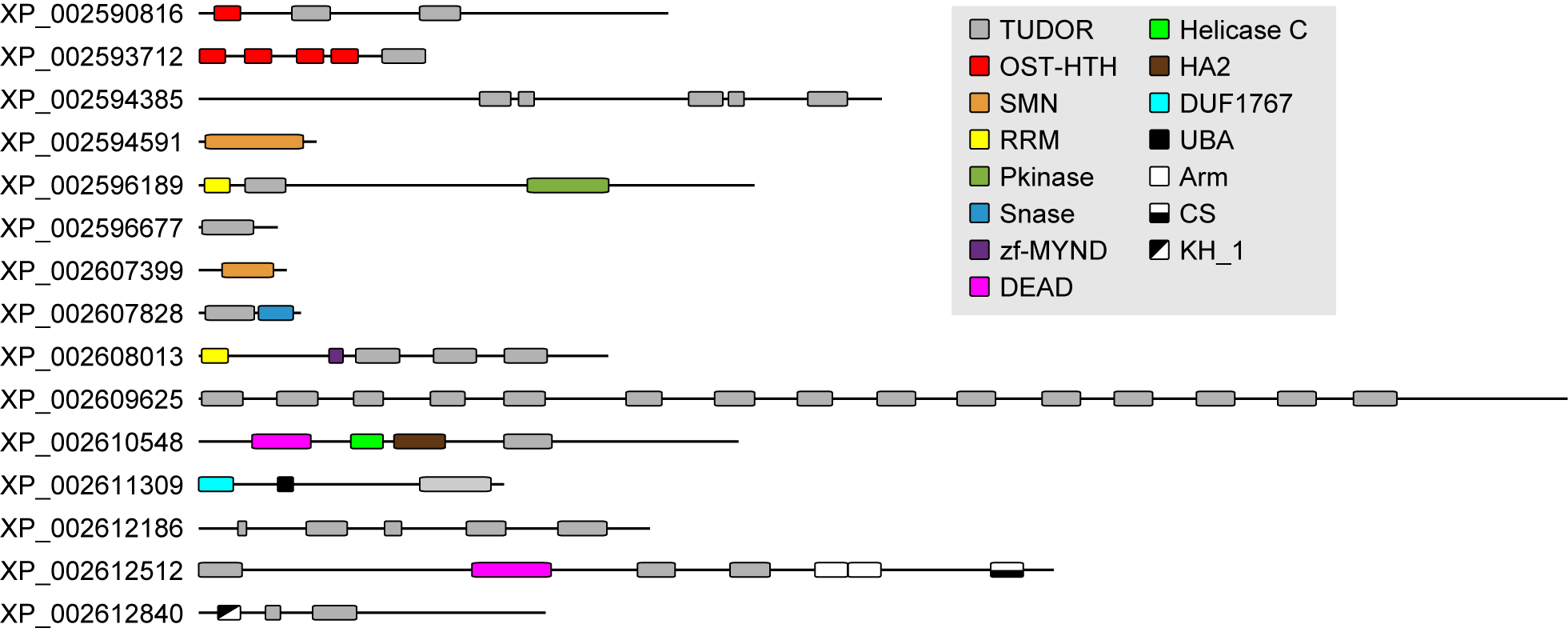

Supplement: Additional file 5: Figure S1 — Schematic depiction of the composition of major domains in B. floridae Tudor domain-containing proteins. [file 2041-9139-4-17-S5.tiff]

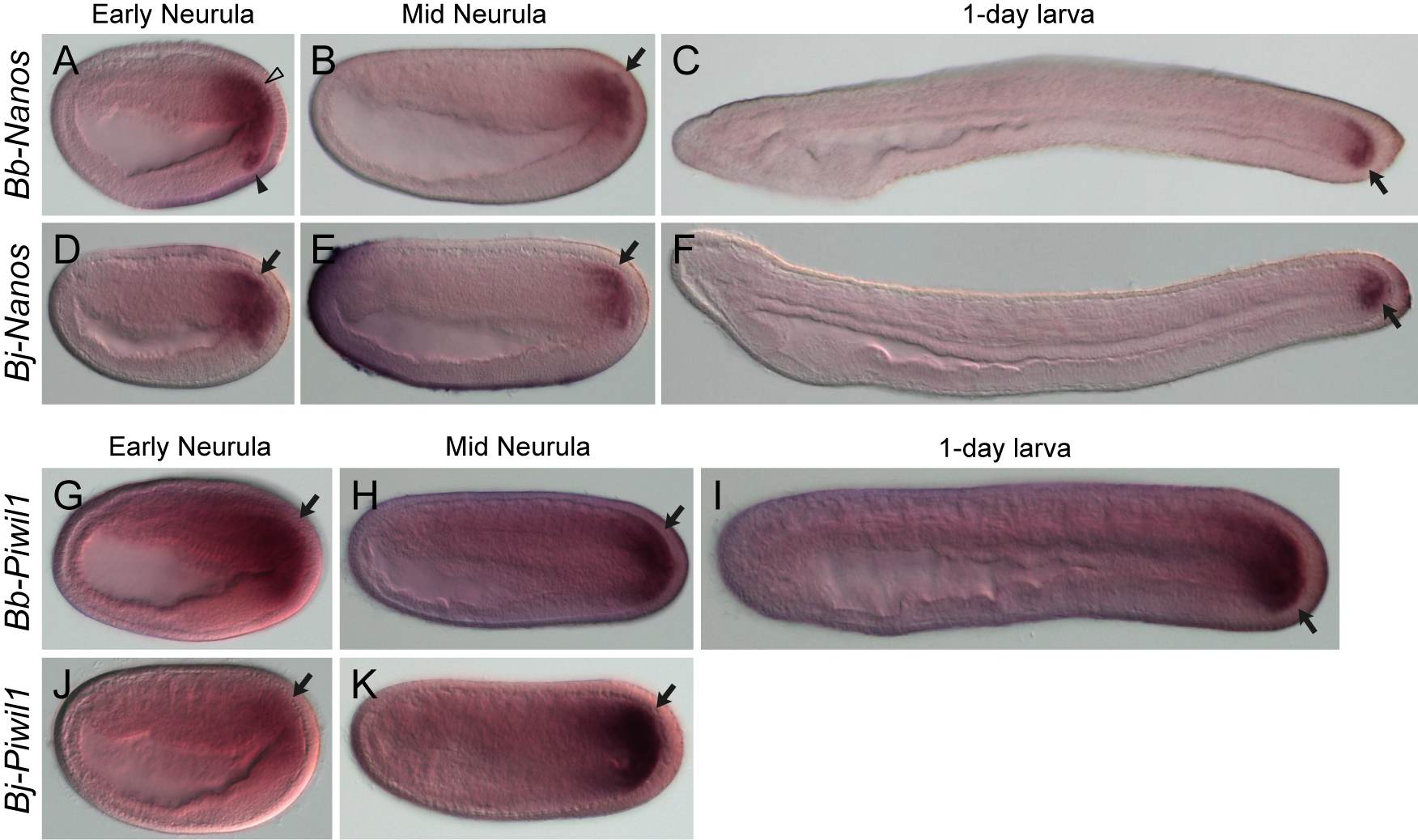

Supplement: Additional file 6: Figure S2 — Expression patterns of Nanos and Piwil1 homologs in B. belcheri and B. japonicum during the neurula and larval stages. Black arrowhead indicates the expression in the putative PGCs, and hollow arrowhead indicates the zygotic expression around the tail bud region. Arrows indicate the merged expression domain in the putative PGCs and the posterior tail bud. [file 2041-9139-4-17-S6.tiff]
